# Pathophysiological Consequences of a Break in S1P1-Dependent Homeostasis of Vascular Permeability Revealed by S1P1 Competitive Antagonism

**DOI:** 10.1371/journal.pone.0168252

**Published:** 2016-12-22

**Authors:** Marc Bigaud, Zuhal Dincer, Birgit Bollbuck, Janet Dawson, Nicolau Beckmann, Christian Beerli, Gina Fishli-Cavelti, Michaela Nahler, Daniela Angst, Philipp Janser, Heike Otto, Elisabeth Rosner, Rene Hersperger, Christian Bruns, Jean Quancard

**Affiliations:** Novartis Institutes for Biomedical Research, Autoimmunity, Transplantation and Inflammation, Basel, Switzerland; Hungarian Academy of Sciences, HUNGARY

## Abstract

**Rational:**

Homeostasis of vascular barriers depends upon sphingosine 1-phosphate (S1P) signaling via the S1P1 receptor. Accordingly, S1P1 competitive antagonism is known to reduce vascular barrier integrity with still unclear pathophysiological consequences. This was explored in the present study using NIBR-0213, a potent and selective S1P1 competitive antagonist.

**Results:**

NIBR-0213 was tolerated at the efficacious oral dose of 30 mg/kg BID in the rat adjuvant-induced arthritis (AiA) model, with no sign of labored breathing. However, it induced dose-dependent acute vascular pulmonary leakage and pleural effusion that fully resolved within 3–4 days, as evidenced by MRI monitoring. At the supra-maximal oral dose of 300 mg/kg QD, NIBR-0213 impaired lung function (with increased breathing rate and reduced tidal volume) within the first 24 hrs. Two weeks of NIBR-0213 oral dosing at 30, 100 and 300 mg/kg QD induced moderate pulmonary changes, characterized by alveolar wall thickening, macrophage accumulation, fibrosis, micro-hemorrhage, edema and necrosis. In addition to this picture of chronic inflammation, perivascular edema and myofiber degeneration observed in the heart were also indicative of vascular leakage and its consequences.

**Conclusions:**

Overall, these observations suggest that, in the rat, the lung is the main target organ for the S1P1 competitive antagonism-induced acute vascular leakage, which appears first as transient and asymptomatic but could lead, upon chronic dosing, to lung remodeling with functional impairments. Hence, this not only raises the question of organ specificity in the homeostasis of vascular barriers, but also provides insight into the pre-clinical evaluation of a potential safety window for S1P1 competitive antagonists as drug candidates.

## Introduction

Sphingosine 1-phosphate (S1P) in blood contributes to the homeostasis of vascular barriers by maintaining a balanced signaling at the level of specific S1P1, S1P2 and S1P3 receptors on the vascular endothelium [1,2,3,4]. On the one side, tonic S1P1 signaling stabilizes the adherence and tight junctions between cells [5,6] and causes persistent activation of eNOS with NO production and activation of the soluble guanylate cyclase to maintain patency of the vessels [6]. On the other side, S1P2 and/or S1P3 signaling is associated with a disruption of adherent junctions and an increase in paracellular permeability [5,7,8]. This has been clearly substantiated by *in vivo* studies demonstrating that selective pharmacological antagonism at the level of S1P1 receptors could disrupt the physiologic S1P-signaling to become S1P2/S1P3-dominant and result in a marked loss of capillary integrity and vascular leakage [9,10]. Similar observations were made with all S1P1-selective competitive antagonists described thus far [11].

Although the molecular mechanisms involved in the vascular leakage resulting from S1P1 competitive antagonism are well understood, little is known about their pathophysiological consequences. Observations *in vivo* have thus far been limited to acute pulmonary edema [9,12,13,14,15], raising important questions concerning the reasons for such an apparent lung selective phenomenon, as well as about its duration and relevance. Hence, the primary goal of the present study was to refine our understanding on the acute *vs* long term impacts of S1P1 competitive antagonism mediated barrier changes with the help of NIBR-0213, a potent and selective S1P1 competitive antagonist which had previously demonstrated lung leakage effect but also good oral efficacy and tolerability in a mouse model of autoimmune disease [15].

The Medicinal Chemistry program that invented NIBR-0213 [14,16] was initiated upon demonstration that the pharmacological activity of FTY720/fingolimod, the well well-established S1P1 agonist now widely approved for the treatment of relapsing-remitting multiple sclerosis (MS) [17], was dependent upon S1P1 receptor internalization/degradation resulting in a so-called “functional antagonism” of receptor signaling, with abrogation of S1P-driven egress of peripheral blood lymphocytes (PBL) from lymph nodes [18,19,20]. This strongly suggested that the inhibitory effects of fingolimod on lymphocyte trafficking might be recapitulated by competitive S1P1 antagonists. A supportive evidence was that selective S1P1 receptor knock-down in T-cells could inhibit lymphocyte egress from lymphoid organs [18,21]. Preclinical proof of concept was finally achieved by showing that NIBR-0213 had comparable therapeutic efficacy vs fingolimod in a mouse model of experimental autoimmune encephalomyelitis (EAE), a model of human MS [15]. A potential safety advantage for competitive S1P1 antagonists was foreseen as, in contrast to S1P1 agonists, they were expected not to activate specifically the G protein-coupled inwardly-rectifying potassium (GIRK/IKAch) channels in cardiomyocytes and consequently to be free of first dose heart rate-lowering effect [22,23]. The lung leakage effect of competitive S1P1 antagonists was however considered as a potential safety liability and a second goal for the present study was to estimate a preclinical therapeutic window for NIBR-0213.

As the intention was to assess the impacts of NIBR-0213 on lung tissues via Magnetic Resonance Imaging (MRI) in the rat, the first mandatory step was to assess tolerability and efficacy of NIBR-0213 in a rat model of autoimmune disease, i.e. adjuvant-induced arthritis (AiA) as a model for human rheumatoid arthritis (RA). In the second step, the acute leakage effects of NIBR-0213 at efficacious doses were assessed using various readouts (protein leakage, MRI changes). The last stage was to assess the prolonged effects of NIBR-0213 after 2 weeks of treatment at pharmacological vs supra-maximal doses.

## Materials and Methods

All animal work was performed according to the Swiss federal law for animal protection and approved by the Veterinary Office Basel. Rats were group-housed (2–3/cage) at room temperature (20–25°C) with relative humidity of 41 to 92%, lighting cycle of 12 hours and standard food/water *ad libitum*.

NIBR-0213 was synthesized as previously described [15] and was formulated either in a 0.5% aqueous methylcellulose solution or a 30% PEG/phosphate buffer. Formulations were stored up to 5 days at room temperature under light protection.

### Adjuvant-induced arthritis model

Wistar rats (male, 220–250 g, Charles River-Germany) were injected intradermally at the base of the tail (100μl) with complete Freunds adjuvant containing M. tuberculosis. In this model, arthritis develops in approximately 11–12 days after injection with the adjuvant, as revealed by a severe swelling of the hind paws [24]. The rats were treated orally with FTY720 at 0.1 mg/kg QD, NIBR-0213 at 30 mg/kg BID, or its vehicle at day-5 or day-9 post-injection of complete Freund’s adjuvant, but before the onset of arthritis (n = 5–10/group). To assess the development of arthritis in each animal, the diameters of both hind paws were measured, in the medial-lateral and anterior-posterior directions, to calculate a mean paw diameter (mØ) at baseline and at termination and evaluate paw swelling as the difference between both values (ΔmØ). The inhibitory effects of NIBR-0213 treatments were expressed as % inhibition vs swelling in vehicle-treated group.

### Effects on peripheral blood Lymphocyte (PBL) counts

Lewis or Wistar rats (males, 220–250 g, Charles River-Germany), were treated orally (2–5 ml/kg) with NIBR-0213 (prepared fresh in a 30% PEG/Buffer solution). Longitudinal blood sampling was performed under anesthesia (5 Vol % isofluran; Forene, Abbott) by sublingual puncture, with ~200 μL/sample in EDTA-coated Eppendorf tubes. All blood cell counts were measured by automated hematology analyzer (ADVIA 120, Siemens).

### Effects on lung endothelial/epithelial permeability

Lewis or Wistar rats (males, 220–250 g, Charles River-Germany), were treated orally with NIBR-0213 and, at 5 hrs post-treatment with NIBR-0213, leakage of plasma proteins in the lungs was assessed using the standard Evans blue dye (EBD)-leakage technique [9]. Briefly, EBD (Fluka) was administered intravenously (20 mg/2 ml NaCl/kg), under anesthesia. One hour later, the rats were exsanguinated under deep anesthesia by incision of the vena cava and the pulmonary vessels perfused via the right-ventricle with 10 ml of saline to remove blood and EBD from the vascular spaces. The lungs were removed *en bloc* and dried at 60°C for 24h.

Dried lungs were weighed and immersed into 2 ml formamide (Sigma) for 24h at 37°C and extracted EBD ([EBD]) from each sample was assessed by spectrophotometry at 620 and 740 nm, to correct for contaminating heme pigments with the following formula: OD620 = OD620—(1.426 x OD740 + 0.030).

The EBD concentrations in lung homogenates were finally estimated with the help of parallel EBD-standard curve.

### MRI monitoring of lung changes

Brown Norway rats (males, 220–250 g, Charles River-Germany) were treated orally for 4 days with NIBR-0213 at 30 mg/kg BID (n = 8) or its vehicle (30% v/v PEG200/phosphate buffer; pH 7.4; n = 4). Treatments were precisely administered at 0, 10, 24, 34, 48, 58, 72, and 82 hrs in a total volume of 2 ml/kg.

For each rat, MRI imaging of the lungs was performed at baseline (prior to treatment) and at 6, 24, 72, 78 and 96 hrs of treatment. During MRI acquisition, rats were anaesthetized (2.5% isoflurane, 2:1 O2/N2O via face mask) and placed in a supine position in a cradle made of Plexiglas. Body temperature was maintained at 37 ± 1°C using warm air and measured via a rectal temperature probe (DM 852, Ellab, Copenhagen). All imaging was performed on spontaneously breathing animals, and neither cardiac nor respiratory triggering was applied. Measurements were carried out with a Biospec 47/40 spectrometer (Bruker Medical Systems, Ettlingen, Germany) operating at 4.7 T, equipped with an actively shielded gradient system capable of generating a gradient of 200 mT/m. The operational software of the scanner was Paravision (Bruker). The parameters of a gradient-echo sequence were chosen with the aim of detecting fluid signals appearing in the lung after an allergen or endotoxin challenge [25,26,27]: repetition time 5.6 ms; echo time 2.7 ms; excitation pulse (gaussian shape) length = 1 ms; flip angle of the excitation pulse approximately 15°; FOV 6x6 cm^2^; matrix size 256x128; slice thickness 1.5 mm. A single transverse slice image was obtained by computing the two-dimensional Fourier transform of the averaged signal from 45 individual image acquisitions and interpolating the data set to 256x256 pixels. There was an interval of 530 ms between individual image acquisitions, resulting in a total acquisition time of 56 s for a single slice. Twenty slices were acquired consecutively.

For image analysis, the volume of high intensity signal was determined using a semi-automatic segmentation procedure described in detail previously [25]. The segmentation parameters were the same for all the analyzed images, chosen to discriminate regions corresponding to high intensity signals. Since the signals from fluid and vessels were of comparable intensities, the volume corresponding to the vessels was assessed on baseline images and then subtracted from the volumes of high intensity signals determined on images acquired after compound administration. Hence, the differential MRI signal volumes are presented in the results.

Immediately after the MRI acquisitions at 6 and 96 hrs, while the animals were still under anesthesia, blood samples (~0.4 ml) were collected in EDTA-coated Eppendorf tubes from the sublingual vein for the hematology readout (using ADVIA 120 analyzer, Siemens; Switzerland) and NIBR-0213 blood levels.

### Two-week treatment study

Rats (220–250 g, HanRcc, Wist; Harlan, The Netherlands) were randomized for the different treatment groups (5 ♂ and 5 ♀, each):

Group 1: Vehicle, 10 ml/kg QD
Group 2: NIBR-0213 at 30 mg/kg QD, 10 ml/kg
Group 3: NIBR-0213 at 100 mg/kg QD, 10 ml/kg
Group 4: NIBR-0213 at 300 mg/kg QD, 10 ml/kg

All treatments lasted 14 days and, during this time, the rats were monitored twice daily for mortality, daily for changes in body weight and clinical signs and weekly for food consumption. Immediate euthanasia (CO2 inhalation followed by exsanguination) was intended in the case of weight loss ≥20% vs value at start of treatment. One spontaneous death unfortunately occurred in Group 4, shortly after treatment on day 10. Prior to death, this rat did not present distinctive clinical manifestations nor severe weight loss (-10.2% in total) and no relevant macroscopic changes were noted at necropsy. On the basis of histopathological examination, the cause of death could be declared as treatment-related and these observations were included in the final analysis (Table 1).

**Table 1:** Microscopic findings in lungs and heart of rats treated with NIBR-0213. Incidence (%) and mean severity (GRADES: 1 = minimal/very few and small; 2 = slight/few/small; 3 = moderate/moderate number and size; 4 = marked/many/large) of the microscopic findings in lungs and heart of rats treated with NIBR-0213. The individual severity GRADES are presented in S4 Table.

	Vehicle		30 mg/kg QD		100 mg/kg QD		300 mg/kg QD	
	M	F	M	F	M	F	M	F
No. of Animals	5	5	4	5	5	5	5	5
LUNGS								
Edema, alveolar (%)	-	-	25	40	20	20	-	40
(Grade)	-	-	2.0	3.0	2.0	3.0	-	3.0
Hemorrhage, alveolar/interstitial (%)	20	-	50	20	60	20	40	-
(Grade)	*1*.*0*	*-*	*1*.*5*	*1*.*0*	*1*.*0*	*1*.*0*	*1*.*0*	*-*
Necrosis, alveolar (%)	-	-	75	80	80	80	-	60
(Grade)	*-*	*-*	*1*.*0*	*1*.*5*	*1*.*8*	*1*.*0*	*-*	*1*.*3*
Thickening, alveolar (%)	-	-	100	100	100	100	100	100
(Grade)	*-*	*-*	*1*.*5*	*1*.*4*	*1*.*8*	*1*.*6*	*1*.*2*	*1*.*6*
Macrophages, alveolar (%)	-	-	100	100	100	100	100	100
(Grade)	*-*	*-*	*2*.*5*	*2*.*8*	*3*.*0*	*3*.*0*	*1*.*6*	*2*.*8*
Fibrotic foci (%)	-	-	75	40	80	80	20	60
(Grade)	*-*	*-*	*1*.*0*	*1*.*0*	*2*.*0*	*1*.*3*	*2*.*0*	*1*.*7*
HEART								
Edema, perivascular (%)	-	-	75	100	100	80	40	40
(Grade)	*-*	*-*	*1*.*7*	*1*.*4*	*1*.*0*	*1*.*8*	*1*.*0*	*1*.*0*
Degeneration, myocytes (%)	-	-	-	40	40	60	20	40
(Grade)	*-*	*-*	*-*	*2*.*0*	*1*.*5*	*1*.*7*	*2*.*0*	*1*.*5*
Fibrosis, interstitial (%)	-	-	-	-	20	20	-	-
(Grade)	*-*	*-*	*-*	*-*	*1*.*0*	*2*.*0*	*-*	*-*

The last day of treatment, blood samples were collected at 0.5, 1, 3, 7 and 24 hrs post-treatment (~250μl in EDTA via tongue vein under anesthesia with Isoflurane [Forene®; Abbott Laboratories S.A., Cham, Switzerland]; no overnight fasting) for toxicokinetics. Blood samples were also collected for hematology (using automated hematology analyzer, ADVIA 120; Siemens) and clinical biochemistry. All blood samples were stored frozen until further processing. At termination, all rats were euthanized (CO2 inhalation followed by exsanguination) for full necropsy. The main organs/tissues were collected, weighed and processed for later histological analysis.

### Plethysmography

Acute impact of NIBR-0213 at 300 mg/kg QD on respiratory functions were assessed, vs vehicle controls, using whole body plethysmography, as previously described [28]. Prior to the study, all male rats allocated to the test (n = 5/group) were acclimatized to the plethysmography chambers in 3 sessions of at least 1 hr duration. During the study, on day 1, rats were placed in plethysmography chambers for about 30 min prior to treatment in order to establish baselines for respiratory rate (breaths/minute), tidal volume (mL) and minute volume (mL). Post-treatment, the rats were immediately placed back in plethysmography chamber for data recording during about 1hr. They then returned to their home cage until the next recording time points (3, 7 and 24 hrs post-treatment). The whole procedure was repeated after 7 days of treatment. Data analysis was performed using the DSI™ software.

### NIBR-0213 blood levels

The blood samples were analyzed for NIBR-0213 as previously described [15]. Briefly, blood samples were thawed at room temperature and processed using a protein precipitation sample preparation. Analysis was performed by LC-MS/MS using electrospray ionization.

### Histology

The animals were necropsied under standard operating procedures. The tissues were placed in 10% buffered formalin, processed, embedded in paraffin wax, sectioned (3–5μm), and stained with hematoxylin and eosin (H&E) for light microscopic examination. A semi-quantitative scoring system was established as 0 = no changes, 1 = minimal, 2 = mild, 3 = moderate and 4 = marked.

### Statistical analysis

Results of MRI studies are expressed as Mean values (± sem) from n individual animals. Anova (Student-Newman-Keuls) tests were performed to compare the MRI signals at different time points, for both groups of animals.

For body and organ weight data, an automated program was used to decide whether parametric or nonparametric group comparisons should be made. This program uses Kolmogorov's test to examine the normality of the data and Bartlett's test to examine the homogeneity of variances. Accordingly, either Dunnett’s test or Student's t-test for parametric group comparisons or Dunn's test or Wilcoxon's test (U-test) for non-parametric group comparisons were used.

No statistical analysis was performed for nominal or ordinal data, e.g. microscopic examination data. In these cases, the data were compared with data recorded in the reference groups or with historical control data.

For the 2-week treatment- study, an ANOVA test was used for overall assessment of parametric data followed, in case of a significant group effect, by Dunnett’s test or Tuckey-Kramer test for group comparisons. For non-parametric data a Kruskal-Wallis test was used for overall assessment followed, in case of a significant group effect, by Dunn’s test for group comparisons. For qualitative data (e.g. clinical signs) a Chisquare test was used for overall assessment followed, in case of significance, by Fisher’s exact test for group comparisons.

All statistical analysis was performed using GraphPad Prism version 5.00 for Windows, (GraphPad Software, San Diego California USA). Statistical significance was assumed at the 5% probability level.

## Results

### NIBR-0213 is well tolerated at an efficacious dose in a rat adjuvant-induced arthritis (AiA) model

In the rat, the intradermal injection, at the base of the tail, of complete Freund’s adjuvant containing heat-killed *Mycobacterium tuberculosis* induces the development of arthritis within approximately 11–12 days, with a progressive swelling of the hind paws. In this model, previous studies demonstrated the beneficial effect of FTY720 at oral doses ≥ 0.1 mg/kg QD [29]. These historical results were reproduced in the present study with oral FTY720 at 0.1 mg/kg QD which significantly reduced peripheral blood lymphocytes (PBL) counts by 80–86% and inhibited paw swelling by 81 ± 7 and 25 ± 10%, when given at day 5 or day 9 post-Freund’s adjuvant, respectively.

**Fig 1:**
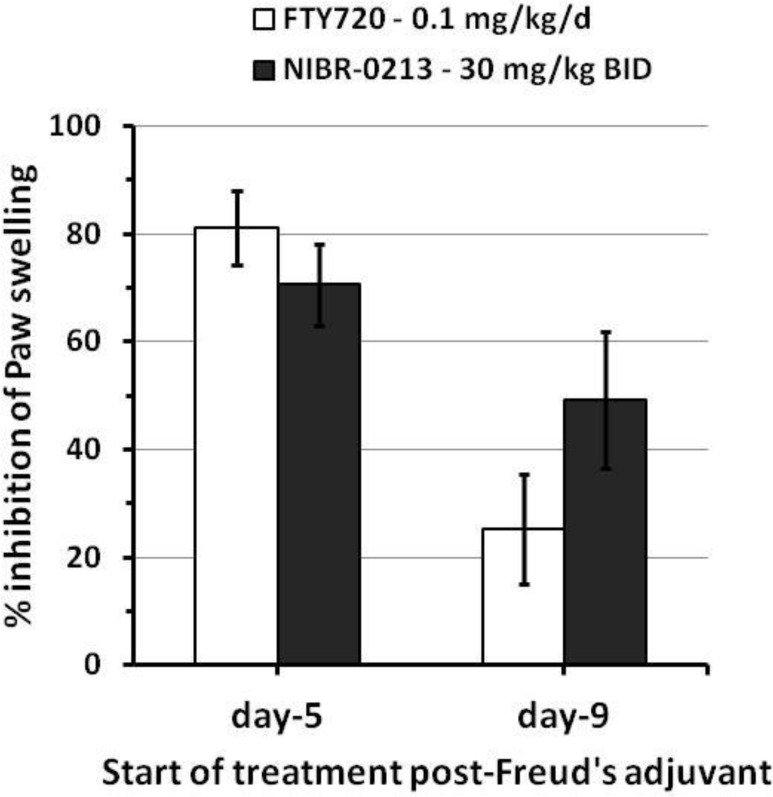
Inhibitory efficacy of NIBR-0213 in a rat AiA model. Inhibition of paw swelling (as measured at day 12 post-adjuvant challenge) in the rat AiA model in response to oral treatments with NIBR-0213 (30 mg/kg BID, n = 10/group) or FTY720 (0.1 mg/kg QD, n = 5/group), starting at either 5 or 9 days post-challenge with Freund Adjuvant. Individual mean mØ and ΔmØ values are presented in S1 Table.

Under these conditions, NIBR-0213, given orally at 30 mg/kg BID, a dose known to reduce PBL counts in the rat by 75–85% over 24 hrs [15], reduced paw swelling by 71 ± 8 and 49 ± 13%, when given at day-5 or day-9 post-Freund’s adjuvant, respectively (Fig 1).

The inhibitory effects of NIBR-0213 at 30 mg/kg BID was not statistically different *vs* FTY720 at 0.1 mg/kg QD. The blood levels of NIBR-0213 were 1.6–1.9 μM at 24 hrs post-dosing. Importantly, NIBR-0213 treatment was well tolerated and no sign of discomfort or breathing problems was observed at any time during the study.

### NIBR-0213 induces marked and dose-dependent acute increase in vascular permeability predominantly in the rat lung

**Fig 2:**
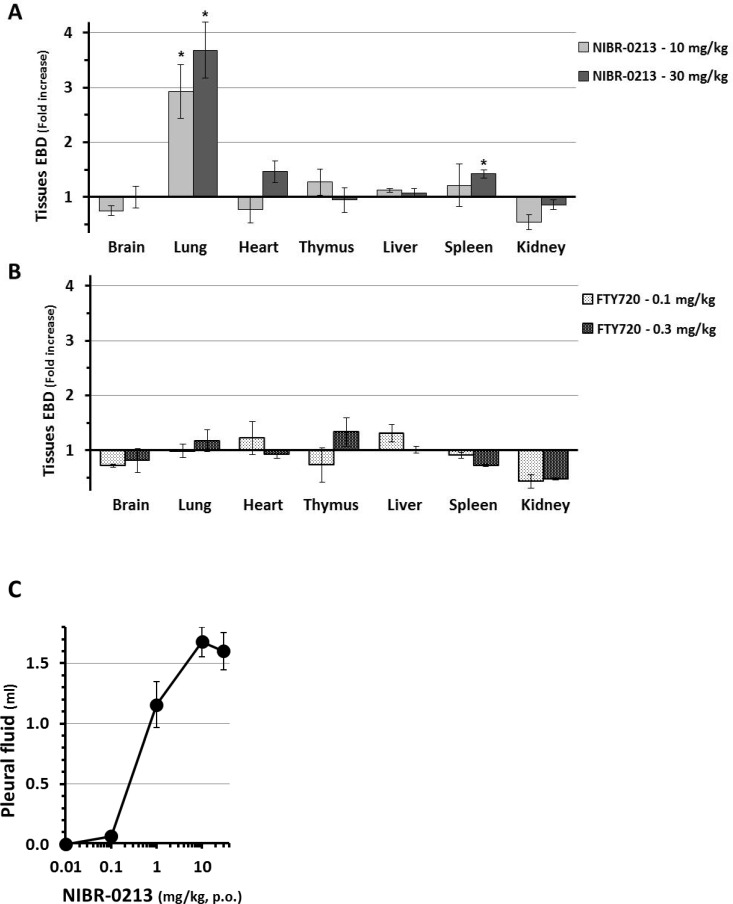
Impacts of NIBR-0213 on vascular permeability in tissues. Evans Blue Dye (EBD) leakage measured in various organs at 6 hrs post-oral treatments with, **A-** NIBR-0213 (10 or 30 mg/kg; n = 3–5 each), **B-** FTY720 (0.1 or 0.3 mg/kg; n = 3 each). Results are expressed as mean ± s.e.m. fold increase *vs* respective vehicle controls. The individual EBD measurements are presented in S2 Table. **C**- Dose-dependent changes in lung pleural fluid measured in rats at 6 hrs post-oral treatments with NIBR-0213 (n = 4–6, each). * p<0.05.

The Evans Blue Dye (EBD) leakage model has been used to demonstrate the induction by S1P1-antagonists of acute plasma protein leakage in the rat lung [9,13,30]. For NIBR-0213, this effect was previously reported to be clearly dose-dependent with, as for its PBL count reduction effect, 50% (ED50) and maximal (Emax) effects obtained at 0.1 mg/kg and ≥ 0.3 mg/kg, respectively [15]. In the present study, the leakage effects of NIBR-0213 in the lungs were compared to those observed in the brain, heart, thymus, liver, spleen and kidney. As illustrated in Fig 2A, 6 hours after NIBR-0213 treatment at 10 or 30 mg/kg, a severe (3–4 fold) increase in EBD leakage was seen in the lungs, whereas only a slight increase (of about 1.5 fold) in EBD leakage was detected in the heart and spleen at 6 hrs post-NIBR-0213 at the highest dose of 30 mg/kg. For comparison, no significant EBD accumulation was observed in any of the organs tested following treatments with efficacious oral doses of FTY720 (0.1 and 0.3 mg/kg) (Fig 2B).

During the process of lung collection for EBD measurements, the amount of pleural fluid found in the thorax of NIBR-0213 treated rats was collected and measured. This indicated that NIBR-0213 elicited a clear dose-dependent increase in pleural fluid, with a maximum of 1.5–2 ml in response to doses ≥ 10 mg/kg and an ED_50_ of ~1 mg/kg (Fig 2C), *i*.*e*. about 10 fold higher when compared to the previously observed ED_50_ for the PBL-reduction and EBD-leakage effects.

Overall, although the lungs was not the only organ affected by the dose-dependent leakage effects of NIBR-0213, it appeared as particularly sensitive and therefore as most suitable to study the dynamic of this phenomenon *in vivo* via Magnetic Resonance Imaging (MRI) monitoring.

### NIBR-0213-induced fluid leakage in the rat lungs is acute and transient as revealed by longitudinal MRI monitoring

In contrast to the EBD-leakage model, MRI is a non-invasive technique that allows the longitudinal monitoring over days of fluid content in tissues of the same animals. Hence, the lungs of rats treated orally during 4 days with NIBR-0213 at 30 mg/kg BID (*i*.*e*. dosing precisely at 0, 10, 24, 34, 48, 58, 72, and 82 hrs of treatment, n = 8) were examined by MRI at baseline and at 6, 24, 72, 78 and 96 hrs of treatment.

Under these conditions, NIBR-0213 blood levels at 6 and 96 hrs of treatment were within the expected ranges, *i*.*e*. 1.3 ± 0.1 and 0.42 ± 0.05 μg/ml, respectively. Also as expected, PBL counts were reduced by 75–80% *vs* baseline, or vehicle controls, throughout the treatment period (results not shown).

**Fig 3:**
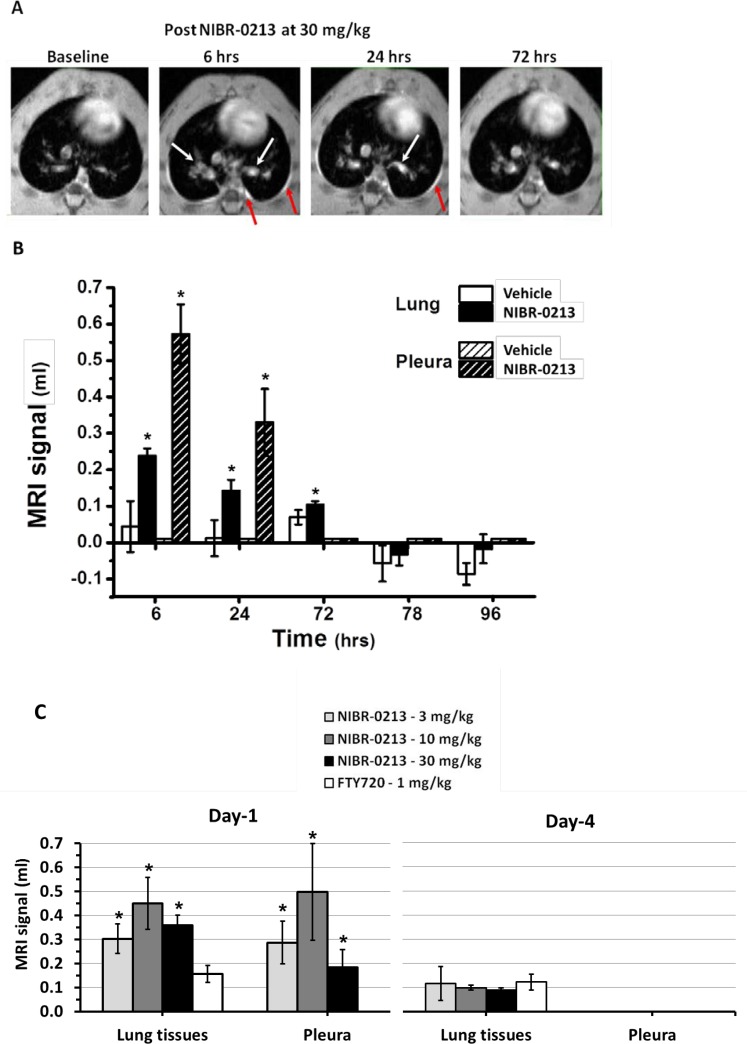
NIBR-0213-induced vascular leakage in the lungs evaluated by MRI. **(A)** Axial MRI sections through the chest of one animal at approximately the same anatomical location acquired before (baseline) and at different time points with respect to beginning of treatment with NIBR-0213 (30 mg/kg BID). The white and red arrows display fluid signals in the lungs and the pleura, respectively, elicited by the compound. **(B)** Differential MRI signal volumes (mean ± s.e.m; n = 4–8) in the lungs and pleura. For the lungs, differential volumes (*i*.*e*. baseline-subtracted) are presented. **(C)** MRI signals (means ± s.e.m, n = 3) in the lungs and pleura at 24 h and 96 h after beginning of treatment with NIBR-0213 (3, 10 or 30 mg/kg BID) or FTY720 (1 mg/kg QD). For the lungs, differential signal volumes (*i*.*e*. baseline-subtracted) are presented. * p<0.05.

A representative example of the lung longitudinal imaging of one NIBR-0213-treated rat is shown in Fig 3A. The white arrows indicate discrete fluid signals appearing in various areas of the lungs, mainly at 6 and 24 hrs. At the same time, marked signals were also detected in the pleura (red arrows). From time point 72 hrs onwards, fluid signals in the lung tissues and pleura had resolved. By contrast, no fluid signals were detected in vehicle-treated animals. The quantitative analysis of the MRI signals (as expressed as fluid volume) is summarized in Fig 3B. Hence, at 6 hrs after starting NIBR-0213 treatment, fluid volume in the lung tissues and pleura could be estimated at about 0.25 ml and 0.6 ml, respectively. At 24 hrs, these volumes were reduced by 30–40% and could no longer be detected after 72 hrs, despite continuation of NIBR-0213 treatment.

The transient nature of the fluid increase in lung tissues and pleura in response to NIBR-0213 was reproduced in a second study using a similar protocol. This time, in order to identify the lowest maximal dose, NIBR-0213 was tested at three doses (30, 10 and 3 mg/kg BID; n = 3 each), all higher than the Emax doses estimated for the acute plasma protein and pleural fluid leakages (0.3 and 1 mg/kg, respectively). As shown in Fig 3C, all three doses triggered similar fluid increases in both lung tissues and pleura compartments at 6 hrs post-treatment, whereas no significant changes were observed after 96 hrs of treatment. Under the same conditions, FTY720 at 1 mg/kg QD did not induce any significant leakage effect up to 96 hrs post-treatment.

Taken together, these results suggest that the dose of 3 mg/kg BID is most likely the lowest Emax dose for the leakage effects of continuous NIBR-0213 treatments in the rat and that, at this dose, these effects are short lived, even in one of the most sensitive organ such as the lung. Hence, powerful regulatory mechanisms could be suspected to counter-balance the leakage effects of S1P1 blockade and to explain why NIBR-0213 is apparently well tolerated at pharmacological doses. As an attempt to overcome those regulatory mechanisms, follow up studies were performed with NIBR-0213 given at supra-maximal doses.

### NIBR-0213 at a supra-maximal dose induced acute pulmonary functional impairments

**Fig 4:**
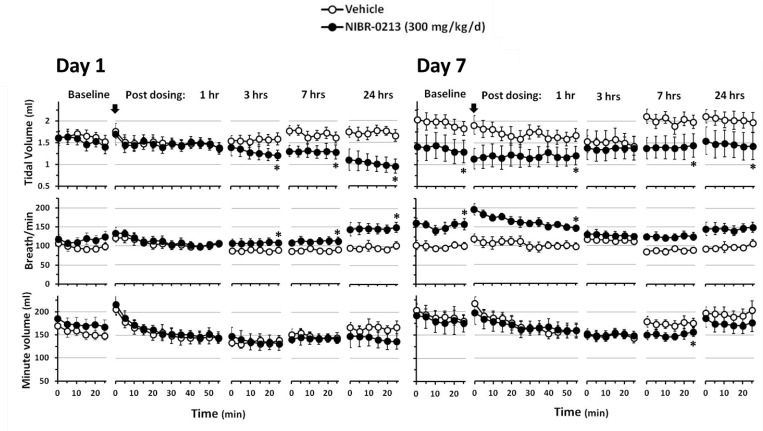
Impacts of NIBR-0213 on lung functions. Whole body plethysmography of male rats treated orally with NIBR-0213 at 300 mg/kg QD, or its vehicle, for 24 hrs (n = 5 each). * p<0.05.

Respiratory parameters were monitored using whole body plethysmography in male rats treated at the supra-maximal dose of 300 mg/kg QD for one week (Fig 4).

Although none of the NIBR-0213 treated-animals showed signs of labored breathing, plethysmography detected the development of a decrease in tidal volume (TV), starting at 3 hrs post-dose and reaching a significant reduction of 41% *vs* controls at 24 hrs post-dose. This was compensated by a parallel increase in breathing rate (Breaths per minute, BPM) so the minute volume (MV) was not significantly affected, indicative of no decrease in oxygen supply. Similar changes were observed after 7-days of NIBR-0213 treatment at baseline, *i*.*e*. just prior to dosing, and no major additional effects vs controls were observed during the 24 hrs post-dose

### Prolonged NIBR-0213 treatments over 2 weeks induces major remodeling in rat lungs

Rats were continuously treated orally over two weeks with NIBR-0213 at two supra-maximal doses (100 and 300 mg/kg QD) and compared to rats treated in parallel with a more pharmacological dose, 30 mg/kg QD, expected to be maximal. Based on previous observations, particular attention was paid to clinical pathology, macroscopic and microscopic changes in the respiratory and cardiovascular systems.

The NIBR-0213 blood levels measured on the last day of the study were, as expected, clearly dose-related in the upper μM range, with 30, 100 and 300 mg/kg QD achieving within 3 hrs 5.5–8.2 μM, 7.1–18.4 μM and 15.3–29.9 μM respectively, and at 24 hrs post treatment 0.1–0.6 μM, 1.4–6.7 μM and 10.3–12.9 μM, respectively. Under these conditions, NIBR-0213 induced severe reduction in PBL counts (-77 to -86%) in all treated animals as well as pronounced signs of clinical pathology with marked macroscopic and microscopic changes, predominantly in the lungs and heart.

**Fig 5:**
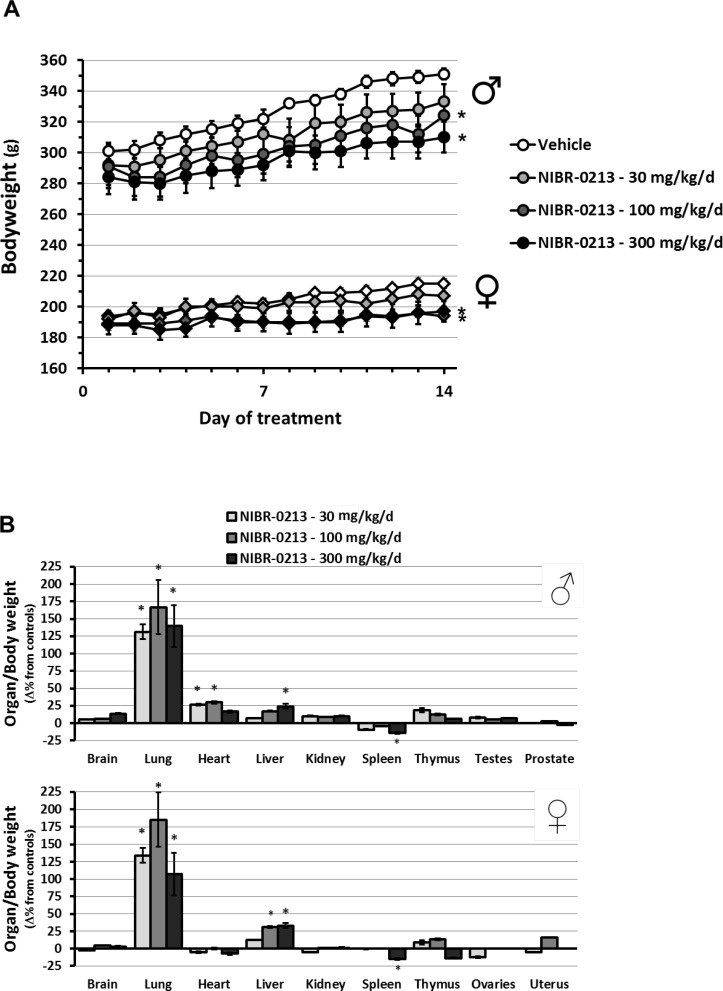
Macroscopic changes provoked by NIBR-0213 treatments in rats. Changes in bodyweight **(A)** and organ weight **(B)** of rats (males or females) treated orally with NIBR-0213 at 30, 100 or 300 mg/kg QD, or its vehicle, during 2 weeks. The mean body and organ weights measured in each groups at termination are presented in S3 Table. Results are mean ± s.e.m. (n = 4–5/group/sex). * p<0.05.

During in-life monitoring, significantly reduced body weight gain was observed in both males and females treated with 100 and 300 mg/kg QD (Fig 5A). Also, labored and/or increased respiration was observed on Day 2 of treatments, then on Day 5 again to continue until the termination of the study. These changes, in line with our previous MRI and plethysmography observations, seemed more pronounced in rats dosed at 300 mg/kg QD. At all doses tested, some clinical pathology changes observed in males and females such as mild increases in neutrophils and/or monocytes were indicative of inflammation.

At necropsy, all NIBR-0213-treated lungs appeared enlarged, pale and/or red (congested) with, in few cases, surrounding foamy outflow. These lungs also showed marked increases in weight relative to total body weight that were similar (about 2 fold) in all treatment groups (Fig 5B). Smaller but significant increases in organ/body weight ratio (~ 25%) were also observed in the heart and liver.

Microscopic examination of the lungs showed a mixture of acute and adaptive changes that were similar, in incidence and severity (varying from minimal to moderate levels) in all NIBR-0213-treatment groups (Table 1).

**Fig 6:**
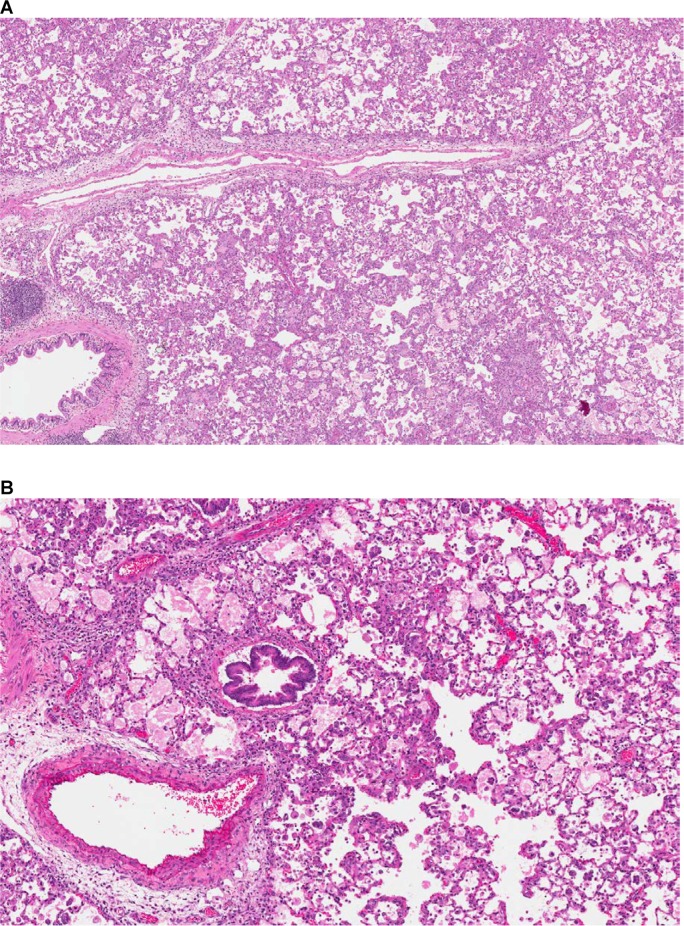
Acute microscopic changes provoked by NIBR-0213 in rat lungs. An overview of acute inflammatory changes (**A:** 4X, H&E) and of perivascular/alveolar/interstitial edema (**B**: 8X, H&E) observed in the lungs of rats treated orally over 2 week with NIBR-0213 at 100 or 300 mg/kg QD, respectively.

**Fig 7:**
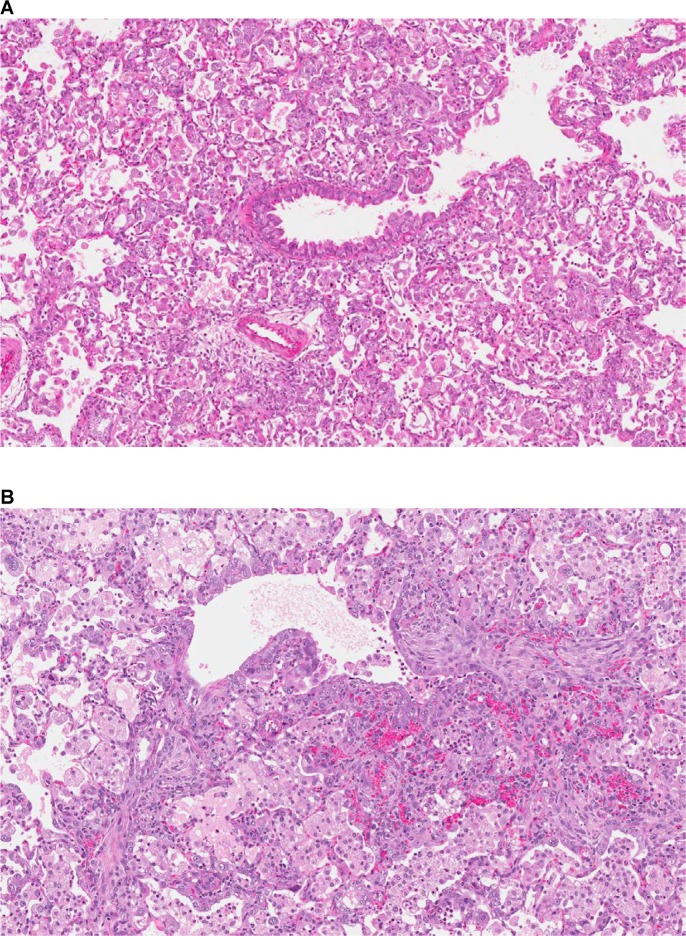
Chronic microscopic changes provoked by NIBR-0213 in rat lungs. An overview of chronic changes with alveolar degeneration /regeneration and accumulation of macrophages (**A:** 10X, H&E.) and of fibrotic foci (**B:** 12X, H&E.) observed in the lungs of a rat treated orally over 2 weeks with NIBR-0213 at 300 and 100 mg/kg QD, respectively.

Acute changes were diffuse alveolar/interstitial hemorrhage, edema, and inflammation, indicative of vascular leakages/damages (Fig 6A and 6B). Chronic changes were the consequences of the hypoxia due to continuous vascular leakage/damage provoked by the NIBR-0213 administration and consisted of: 1- Alveolar degenerative (necrosis) and regenerative (wall thickening characterized by type II pneumocytes hypertrophy/hyperplasia); 2- Accumulation of macrophages with enlarged and foamy cytoplasm, and multinucleated appearance (Fig 7A); 3- fibrotic foci considered as the final stage of repair/remodeling process (Fig 7B).

**Fig 8:**
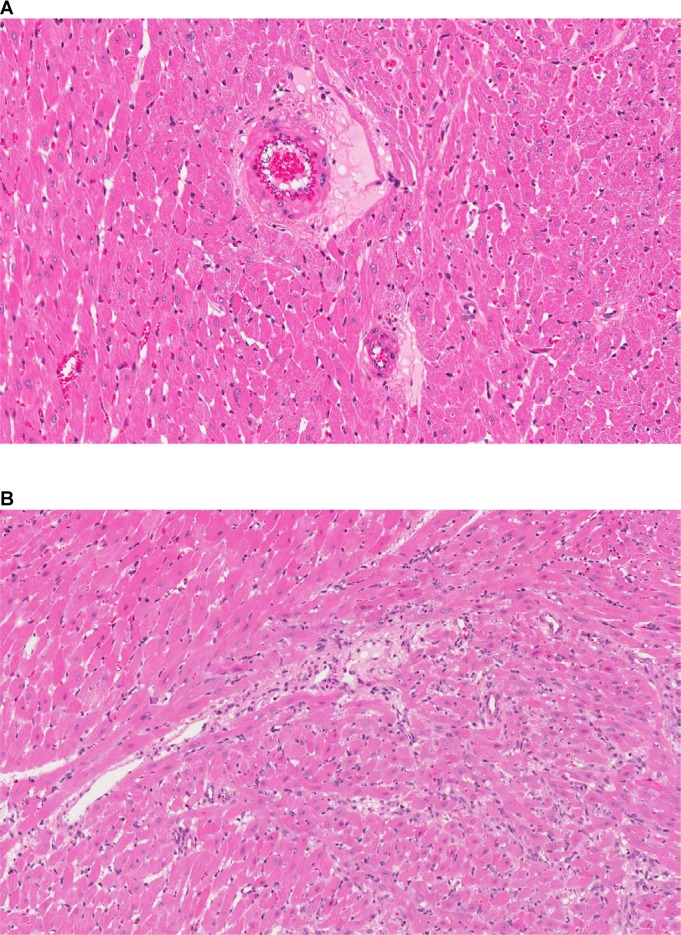
Acute microscopic changes provoked by NIBR-0213 in rat hearts. Perivascular edema (**A:** 20X, H&E.) and focal degenerative myocytes with interstitial inflammatory cells and fibrosis (**B:** 20X, H&E.) observed in the hearts of a rat treated orally over 2 weeks with NIBR-0213 at 100 mg/kg QD.

In addition, changes indicative of major vascular leakage/damage were also observed in the heart. They were characterized by minimal to mild perivascular edema and degenerative myocytes associated with cellular debris and inflammatory cell infiltrates present particularly at the base of the heart (Fig 8A). Interstitial fibrotic foci were also noted in few animals dosed at 100 mg/kg QD (Fig 8B).

## Discussion

The main objective of the present study was to explore *in vivo* the functional/ pathophysiological consequences of a break in the S1P1-dependent vascular permeability homeostasis provoked by S1P1 competitive antagonism. For this, the approach was to explore in the rat the various impacts of treatments with NIBR-0213, a potent and selective S1P1 competitive antagonist with good oral efficacy [15], first at a tolerated and fully efficacious dose then at supra-maximal doses.

We previously reported that NIBR-0213 was tolerated and near maximally efficacious at 30 mg/kg BID in a validated mouse model of autoimmune disease [15]. The results of the present study confirm that NIBR-0213 at this dose is also tolerated and as efficacious as FTY720 in a rat model of autoimmune disease (adjuvant-induced arthritis). This is in line with a previous study showing beneficial effects of another competitive S1P1 antagonist (TASP0277308) in a mouse arthritis model, with reduction of T cell infiltration into the joints and modulation of local cytokine production [12]. However, concerning the apparent tolerability of NIBR-0213 at efficacious doses (with no obvious sign of discomfort and/or changes in breathing behavior), it is intriguing in the light of the significant and dose-dependent acute vascular leakage effects that were observed in various organs, but mostly in the lungs, of NIBR-0213-treated rats. Similar drug-induced leakages were previously reported for NIBR-0213 [15] as well as for all the competitive S1P1 antagonists described thus far [9,11,13]. Hence, we performed a deeper analysis of NIBR-0213-induced lung leakage in order to assess the pathophysiological relevance of S1P1 competitive antagonism-induced increase vascular permeability, confirm or not the apparent tolerability of NIBR-0213 and ultimately identify potential safety liabilities for competitive S1P1-antagonists.

With the use of longitudinal MRI monitoring, the present study clearly demonstrates that the impacts on lung leakage of NIBR-0213 at a nearly maximally efficacious dose are acute and transient, appearing within the first 6 hrs upon initial application and resolving within 3–4 days of continuous treatment. Also, they appear quite complex, as combining severe increases in local vascular permeability together with marked and dose-related pleural effusion. This suggests for the first time that tonic S1P1 signaling is involved in the maintenance of the pleural integrity in addition to its well established role in the control of vascular permeability [1,2,3,4]. The pathophysiological mechanisms that regulate pleural fluid and the permeability of the pleural mesothelium have been well studied [31,32] but, to our knowledge, a role for the S1P/S1P1 axis has not been described as yet. Hence, follow up work is required for a better understanding of the S1P1-dependent control of pleural permeability. But this could already be seen as a potential amplifying factor for the S1P1 competitive antagonism-induced acute and transient vascular leakage and, therefore, as a putative explanation for the remarkably high sensitivity of lungs vs other organs. In addition, the absence of any detectable acute impact on the lung functions strongly suggests that, as in human, acute pleural effusion is mostly asymptomatic in the rat and needs a certain level of intensity and/or chronicity to become apparent [33,34].

Concerning the needs for high intensity for S1P1 competitive antagonism-induced lung/pleural leakage to impact the respiratory function, it is supported in the present study by the observation that supra-maximal NIBR-0213 doses are required to provoke measurable signs of acute lung failure within few hours upon dosing. About the needs for chronicity, this is also supported in the present study by the development, after 2 weeks of NIBR-0213-treatments, of severe tissue remodeling hallmarks of local inflammation in lungs (increases in alveolar wall thickening, fibrosis, macrophage infiltration, hemorrhage, edema and focal necrosis), as well as in other organs such as heart.

Taken altogether, these observations strongly suggest that, although asymptomatic and transient, the acute lung specific leakage effects provoked by the blockade of the S1P1 receptors by NIBR-0213 are associated with the development of chronic lung inflammatory remodeling that could remain silent for a while, but could ultimately precipitate loss of lung function, as seen in an accelerated and amplified way under NIBR-0213 at a supra-maximal dose. The underlying mechanisms of such a chronic lung inflammatory remodeling are currently unknown. A direct consequence of S1P1 competitive antagonism on lung fibrosis/remodeling could be an option, as previously seen in a model of pulmonary injury [35]. An indirect consequence of the S1P1-dependent lung vascular/pleural leakage effects could also be envisioned, as extravasation of plasma proteins into the alveolar space is known to trigger the intra-alveolar activation of the coagulation cascade leading to local fibrosis [36]. Unopposed S1P2- and S1P3-dependent could play a role as well [9,10]. Parallel unspecific toxic effects for NIBR-0213 should not be excluded, especially in the case of supra-maximal doses with negative impacts on animal growth, although their influence on tissues remodeling is most likely limited, as suggested by the fact that the tissues changes provoked by NIBR-0213 at the lowest dose-tested appeared maximal, with no further increase (neither in incidence nor in severity grade) at higher doses. Ultimate discrimination between specific and unspecific effects would require further benchmarking with other S1P1 competitive antagonists.

Overall, the present study suggests that, in the rat, specific competitive S1P1 antagonism-induced disruption of S1P1-dependent homeostasis of vascular permeability is associated with a concomitant disruption in the S1P1-dependent control of pleural permeability. Further, it also suggests that both phenomena combined trigger the development of pathophysiological consequences particularly pronounced in the lungs, but also detectable in the heart, with a first transient and asymptomatic phase of local fluid leakage, followed by a chronic phase of local inflammation and tissue remodeling with gradual loss of function. Ultimately, these effects should be considered as potential safety liabilities for S1P1-competitive antagonists and help estimating preclinical therapeutic index *vs* efficacy in various disease models.

In the case of NIBR-0213 in autoimmune pathologies, there is clearly no therapeutic index in the rat, as both efficacy and lung liabilities were observed at the same doses. It is however still unclear whether or not this is species-dependent, as well as compound- or class-related. Hence, assessing the preclinical safety profile of NIBR-0213 in a higher species such as dog or mini-pig appears as a key mandatory step before considering its further development for autoimmune diseases such as MS or RA. Nevertheless, even in the case of a good therapeutic index in this second species, translation to human would not be possible before specific biomarker(s) for lung leakage/remodeling become available. This conclusion could most likely be extended to the development of all competitive S1P1 antagonists in autoimmune diseases. Another potential indication to be explored would be chemotherapy-induced neuropathic pain, as several S1P1 agonists and a weak competitive S1P1 antagonist have recently demonstrated strong efficacy at unexpectedly low doses in a relevant animal model [37].

## Supporting Information

S1 Table Inhibitory effects of NIBR-0213 (30 mg/kg, BID) and of FTY720 (0.1 mg/kg, QD) in the rat AiA model. Individual mean paw diameters (mØ) measured in rats at baseline and then 12 days post-adjuvant challenge (arthritic). For each rat, each mØ value is the mean of both hind paws and paw swelling (ΔmØ) is evaluated as the difference mØ_Baseline_-mØ_arthritic_. The inhibitory effects of NIBR-0213 treatments, started at either 5 or 9 days post-adjuvant challenge, are calculated as % inhibition vs swelling in vehicle-treated group. * p<0.05
(DOC)

S2 Table Impacts of NIBR-0213 on vascular permeability in organs. Individual Evans Blue Dye leakages ([EBD]) measured in various organs at 6 hrs post-oral treatments with NIBR-0213 (10 or 30 mg/kg), FTY720 (0.1 or 0.3 mg/kg) or vehicle (30% PEG/phosphate buffer). The impact of treatments are evaluated as fold increases vs vehicle controls (treatment groups performed in parallel are indicated as ^1,2^ or ^3^). * p<0.05.
(DOC)

S3 Table Macroscopic changes provoked by NIBR-0213 treatments in rats. Mean body and organ weights in rats after 2 weeks of oral treatment with NIBR-0213 at 30, 100 or 300 mg/kg QD, or its vehicle. Results are mean ± s.e.m (n = 10/group/sex). *p<0.05
(DOC)

S4 Table Microscopic findings in lungs and heart of rats treated with NIBR-0213 over 2 weeks. Individual severity GRADES (- = none; 1 = minimal/very few and small; 2 = slight/few/small; 3 = moderate/moderate number and size; 4 = marked/many/ large) of the microscopic findings in lungs and heart of rats treated with NIBR-0213 or its vehicle. For each group, the Grades are indicated under the same the rat-order.
(DOC)
