# Supplementary material for: Pathophysiological Consequences of a Break in S1P1-Dependent Homeostasis of Vascular Permeability Revealed by S1P1 Competitive Antagonism
Source: PLoS One. 2016 Dec 22;11(12):e0168252. doi: 10.1371/journal.pone.0168252 (PMC5179015; doi:10.1371/journal.pone.0168252)
Supplement: S1 Table — Individual mean paw diameters (mØ) measured in rats at baseline and then 12 days post-adjuvant challenge (arthritic). For each rat, each mØ value is the mean of both hind paws and paw swelling (ΔmØ) is evaluated as the difference mØBaseline-mØarthritic. The inhibitory effects of NIBR-0213 treatments, started at either 5 or 9 days post-adjuvant challenge, are calculated as % inhibition vs swelling in vehicle-treated group. * p<0.05 (DOC) [file pone.0168252.s001.doc]

**S1 Table. Inhibitory effects of NIBR-0213 (30 mg/kg, BID) and of FTY720 (0.1 mg/kg, QD) in the rat AiA model**

Individual mean paw diameters (mØ) measured in rats at baseline and 12 days post-adjuvant challenge (arthritic). For each rat, each mØ value is the mean of both hind paws and paw swelling (ΔmØ) is evaluated as the difference mØ_Baseline_-mØ_arthritic_ . The inhibitory effects of NIBR-0213 treatments, started at either 5 or 9 days post-adjuvant challenge, are calculated as % inhibition vs swelling in vehicle-treated group. * p<0.05

|  | **Vehicle-treated** | | | **NIBR-0213-treated from day 5** | | | **NIBR-0213-treated from day 9** | | |
| --- | --- | --- | --- | --- | --- | --- | --- | --- | --- |
|  | **mØ_Baseline_**  **(mm)** | **mØ_arthritic_**  **(mm)** | **Swelling (ΔmØ)**  **(mm)** | **mØ_arthritic_**  **(mm)** | **Swelling (ΔmØ)**  **(mm)** | **% Inhibition** | **mØ_arthritic_**  **(mm)** | **Swelling (ΔmØ)**  **(mm)** | **% Inhibition** |
|  | **5.6**  **5.5**  **5.1**  **4.9**  **5.8**  **5.2**  **5.7**  **5.5**  **5.6**  **5.5** | **15.1**  **10.8**  **13.3**  **10.7**  **11.8**  **8.6**  **14.2**  **11.5**  **14.2**  **11.8** | **9.6**  **5.3**  **7.9**  **5.3**  **6.4**  **3.1**  **8.8**  **6.1**  **8.7**  **6.4** | **10.0**  **8.6**  **6.7**  **5.9**  **7.8**  **7.1**  **5.7**  **5.5**  **9.6**  **7.4** | **4.5**  **3.2**  **1.2**  **0.4**  **2.4**  **1.6**  **0.3**  **0.1**  **4.2**  **1.9** | **33.0**  **53.0**  **81.8**  **93.6**  **64.8**  **75.9**  **95.9**  **98.8**  **38.2**  **71.5** | **7.3**  **6.1**  **11.7**  **9.9**  **9.1**  **7.6**  **14.3**  **10.9**  **6.0**  **5.8** | **1.9**  **0.6**  **6.3**  **4.4**  **3.6**  **2.2**  **8.8**  **5.5**  **0.6**  **0.3** | **72.2**  **90.7**  **7.1**  **34.5**  **46.3**  **67.8**  **-30.6**  **18.9**  **91.4**  **95.1** |
| **Mean** | **5.4 ± 0.3** | **12.2 ± 1.9** | **6.8 ± 0.6** | **7.4 ± 1.5** | **2.0 ± 1.5 *** | **70.6 ± 7.4** | **8.8 ± 0.9** | **3.4 ± 0.9 *** | **49.3 ± 13.2** |

|  | **Vehicle-treated** | | | **FTY720-treated from day 5** | | | **FTY720-treated from day 9** | | |
| --- | --- | --- | --- | --- | --- | --- | --- | --- | --- |
|  | **mØ_Baseline_**  **(mm)** | **mØ_arthritic_**  **(mm)** | **Swelling (ΔmØ)**  **(mm)** | **mØ_arthritic_** | **Swelling (ΔmØ)**  **(mm)** | **% Inhibition** | **mØ_arthritic_**  **(mm)** | **Swelling (ΔmØ)**  **(mm)** | **% Inhibition** |
|  | **5.3**  **5.6**  **6.2**  **6.0**  **5.5** | **12.8**  **13.8**  **11.8**  **12.4**  **14.0** | **7.1**  **8.1**  **6.1**  **6.7**  **8.2** | **6.1**  **6.1**  **7.3**  **6.2**  **9.9** | **0.4**  **0.3**  **1.6**  **0.4**  **4.1** | **94.6**  **95.6**  **78.0**  **94.3**  **43.0** | **13.3**  **9.0**  **8.0**  **11.9**  **12.8** | **7.5**  **3.3**  **2.3**  **6.1**  **7.1** | **-4.4**  **54.8**  **68.9**  **15.0**  **2.2** |
| **Mean** | **5.7 ± 0.2** | **13.0 ± 0.4** | **7.2 ± 0.4** | **7.1 ± 0.5** | **1.4 ± 0.5 *** | **81.1 ± 6.7** | **11.0 ± 0.7** | **5.3 ± 0.7 *** | **25.3 ± 10.1** |
