# Supplementary material for: Pathophysiological Consequences of a Break in S1P1-Dependent Homeostasis of Vascular Permeability Revealed by S1P1 Competitive Antagonism
Source: PLoS One. 2016 Dec 22;11(12):e0168252. doi: 10.1371/journal.pone.0168252 (PMC5179015; doi:10.1371/journal.pone.0168252)
Supplement: S2 Table — Individual Evans Blue Dye leakages ([EBD]) measured in various organs at 6 hrs post-oral treatments with NIBR-0213 (10 or 30 mg/kg), FTY720 (0.1 or 0.3 mg/kg) or vehicle (30% PEG/phosphate buffer). The impact of treatments are evaluated as fold increases vs vehicle controls (treatment groups performed in parallel are indicated as 1,2 or 3). * p<0.05. (DOC) [file pone.0168252.s002.doc]

**S2 Table. Impacts of NIBR-0213 on vascular permeability in organs.**

Individual Evans Blue Dye leakages ([EBD]) measured in various organs at 6 hrs post-oral treatments with NIBR-0213 (10 or 30 mg/kg), FTY720 (0.1 or 0.3 mg/kg) or vehicle (30% PEG/phosphate buffer). The impact of treatments are evaluated as fold increases vs vehicle controls (treatment groups performed in parallel are indicated as ^1,2^ or ^3^). * p<0.05.

| **Organ** | **Brain** | | **Lung** | | **Heart** | | **Thymus** | | **Liver** | | **Spleen** | | **Kidney** | |
| --- | --- | --- | --- | --- | --- | --- | --- | --- | --- | --- | --- | --- | --- | --- |
|  | **[EBD]**  **nm** | **Fold increase** | **[EBD]**  **nm** | **Fold increase** | **[EBD]**  **nm** | **Fold increase** | **[EBD]**  **nm** | **Fold increase** | **[EBD]**  **nm** | **Fold increase** | **[EBD]**  **nm** | **Fold increase** | **[EBD]**  **nm** | **Fold increase** |
| **Vehicle^1^** | **0.145**  **0.102**  **0.163** |  | **0.445**  **0.238**  **0.380** |  | **0.438**  **0.552**  **0.925** |  | **0.132**  **0.090**  **0.068** |  | **0.101**  **0.170**  **0.228** |  | **0.257**  **0.180**  **0.282** |  | **0.632**  **0.930**  **0.317** |  |
| **Mean** | **0.137** |  | **0.354** |  | **0.638** |  | **0.097** |  | **0.166** |  | **0.240** |  | **0.626** |  |
| **NIBR0213^1^**  **10 mg/kg** | **0.092**  **0.088**  **0.126** | **0.67**  **0.64**  **0.92** | **0.699**  **1.164**  **1.253** | **1.98**  **3.29**  **3.54** | **0.364**  **0.304**  **0.809** | **0.57**  **0.48**  **1.27** | **0.131**  **0.079**  **0.156** | **1.36**  **0.82**  **1.62** | **0.196**  **0.191**  **0.174** | **1.18**  **1.15**  **1.05** | **0.179**  **0.477**  **0.215** | **0.75**  **1.99**  **0.90** | **0.397**  **0.185**  **0.439** | **0.63**  **0.30**  **0.70** |
| **Mean** | **0.102** | **0.8 ± 0.1** | **1.039** | **2.9 ± 0.5*** | **0.492** | **0.8 ± 0.3** | **0.122** | **1.3 ± 0.2** | **0.187** | **1.1 ± 0.1** | **0.290** | **1.2 ± 0.4** | **0.340** | **0.5 ± 0.1** |
| **FTY720^1^**  **0.1 mg/kg** | **0.082**  **0.149**  **0.101** | **0.60**  **1.09**  **0.74** | **0.369**  **0.495**  **0.346** | **1.04**  **1.40**  **0.98** | **0.645**  **1.090**  **0.617** | **1.01**  **1.71**  **0.97** | **0.053**  **0.075**  **0.117** | **0.55**  **0.77**  **1.21** | **0.210**  **0.238**  **0.156** | **1.27**  **1.43**  **0.94** | **0.197**  **0.268**  **0.218** | **0.82**  **1.12**  **0.91** | **0.270**  **0.465**  **0.315** | **0.43**  **0.74**  **0.50** |
| **Mean** | **0.111** | **0.8 ± 0.1** | **0.403** | **1.1 ± 0.1** | **0.784** | **1.2 ± 0.2** | **0.082** | **0.8 ± 0.2** | **0.201** | **1.2 ± 0.1** | **0.228** | **0.9 ± 0.1** | **0.350** | **0.6 ± 0.1** |
| **Vehicle^2^** | **0.080**  **0.055**  **0.050** |  | **0.274**  **0.180**  **0.137** |  | **0.042**  **0.039**  **0.028** |  | **0.039**  **0.032**  **0.031** |  | **0.074**  **0.052**  **0.040** |  | **0.077**  **0.082**  **0.083** |  | **0.148**  **0.074**  **0.068** |  |
| **Mean** | **0.062** |  | **0.197** |  | **0.036** |  | **0.034** |  | **0.055** |  | **0.081** |  | **0.097** |  |
| **Vehicle^3^** | **0.116**  **0.115**  **0.184** |  | **0.338**  **0.290**  **0.222** |  | **0.440**  **0.283**  **0.287** |  | **0.334**  **0.073**  **0.106** |  | **0.116**  **0.115**  **0.184** |  | **0.174**  **0.158**  **0.189** |  | **0.807**  **0.911**  **0.918** |  |
| **Mean** | **0.138** |  | **0.283** |  | **0.337** |  | **0.171** |  | **0.138** |  | **0.174** |  | **0.879** |  |
| **NIBR0213^2^**  **30 mg/kg** | **-**  **0.043**  **0.060** | **-**  **0.69**  **0.97** | **0.741**  **1.035**  **0.715** | **3.76**  **5.26**  **3.63** | **0.055**  **0.038**  **0.069** | **1.52**  **1.04**  **1.89** | **0.030**  **0.053**  **0.041** | **0.88**  **1.56**  **1.21** | **0.052**  **0.052**  **0.066** | **0.94**  **0.94**  **1.19** | **0.132**  **0.115**  **0.115** | **1.64**  **1.43**  **1.43** | **0.064**  **0.076**  **0.090** | **0.66**  **0.79**  **0.93** |
| **NIBR0213^3^**  **30 mg/kg** | **-**  **0.202**  **0.103** | **-**  **1.46**  **0.74** | **-**  **0.693**  **0.939** | **-**  **2.45**  **3.32** | **-**  **0.672**  **0.581** | **-**  **2.00**  **1.73** | **-**  **0.093**  **0.090** | **-**  **0.54**  **0.53** | **-**  **0.146**  **0.170** | **-**  **1.06**  **1.23** | **-**  **0.227**  **0.228** | **-**  **1.31**  **1.31** | **-**  **0.763**  **0.934** | **-**  **0.87**  **1.06** |
| **Mean** | **0.102** | **1.0 ± 0.2** | **0.825** | **3.7 ± 0.4*** | **0.283** | **1.6 ± 0.2*** | **0.061** | **0.9 ± 0.2** | **0.097** | **1.1 ± 0.1** | **0.163** | **1.4 ± 0.1*** | **0.385** | **0.9 ± 0.1** |
| **FTY720^3^**  **0.3 mg/kg** | **0.117**  **0.146**  **0.096** | **0.85**  **1.06**  **0.70** | **0.290**  **0.396**  **0.289** | **1.02**  **1.40**  **1.02** | **0.414**  **0.326**  **0.251** | **1.23**  **0.97**  **0.74** | **0.191**  **0.301**  **0.167** | **1.12**  **1.76**  **0.98** | **0.123**  **0.148**  **0.181** | **0.89**  **1.07**  **1.31** | **0.170**  **0.158**  **0.113** | **0.98**  **0.91**  **0.65** | **0.293**  **0.633**  **0.541** | **0.33**  **0.72**  **0.62** |
| **Mean** | **0.120** | **0.9 ± 0.1** | **0.325** | **1.2 ± 0.1** | **0.330** | **1.0 ± 0.1** | **0.220** | **1.3 ± 0.2** | **0.151** | **1.1 ± 0.1** | **0.147** | **0.8 ± 0.1** | **0.489** | **0.6 ± 0.1** |
