# Supplementary material for: Pathophysiological Consequences of a Break in S1P1-Dependent Homeostasis of Vascular Permeability Revealed by S1P1 Competitive Antagonism
Source: PLoS One. 2016 Dec 22;11(12):e0168252. doi: 10.1371/journal.pone.0168252 (PMC5179015; doi:10.1371/journal.pone.0168252)
Supplement: S3 Table — Mean body and organ weights in rats after 2 weeks of oral treatment with NIBR-0213 at 30, 100 or 300 mg/kg QD, or its vehicle. Results are mean ± s.e.m (n = 10/group/sex). *p<0.05 (DOC) [file pone.0168252.s003.doc]

**S3 Table. Macroscopic changes provoked by NIBR-0213 treatments in rats.**

Mean body and organ weights in rats after 2 weeks of oral treatment with NIBR-0213 at 30, 100 or 300 mg/kg QD, or its vehicle. Results are mean ± s.e.m (n=10/group/sex). *p<0.05

|  |  | **NIBR-0213 (mg/kg/d; QD)** | | |
| --- | --- | --- | --- | --- |
| **Weight (g)** | **Vehicle** | **30** | **100** | **300** |
| **Males** |  |  |  |  |
| Whole Body | 348 ± 5 | 325 ± 11 | 322 ± 11 | 303 ± 12 |
| Brain | 2.1 ± 0.1 | 2.0 ± 0.1 | 2.0 ± 0.1 | 2.0 ± 0.1 |
| Lung | 1.7 ± 0.2 | 3.6 ± 0.2 * | 4.1 ± 0.5 * | 3.5 ± 0.4 * |
| Heart | 1.04 ± 0.03 | 1.25 ± 0.06 * | 1.24 ± 0.05 * | 1.08 ± 0.09 |
| Liver | 13.8 ± 0.4 | 13.8 ± 0.7 | 14.9 ± 0.8 | 15.0 ± 1.3 * |
| Kidney | 2.4 ± 0.1 | 2.4 ± 0.1 | 2.4 ± 0.1 | 2.3 ± 0.1 |
| Spleen | 0.75 ± 0.06 | 0.66 ± 0.03 | 0.69 ± 0.02 | 0.57 ± 0.04 * |
| Thymus | 0.56 ± 0.04 | 0.63 ± 0.04 | 0.58 ± 0.04 | 0.50 ± 0.04 |
| Testes | 3.7 ± 0.1 | 3.7 ± 0.2 | 3.6 ± 0.2 | 3.4 ± 0.1 |
| Prostate | 0.69 ± 0.09 | 0.66 ± 0.04 | 0.60 ± 0.06 | 0.49 ± 0.06 |
| **Females** |  |  |  |  |
| Whole Body | 212 ± 6.5 | 205 ± 10 | 194 ± 5 | 194 ± 10 |
| Brain | 1.9 ± 0.1 | 1.8 ± 0.1 | 1.9 ± 0.1 | 1.8 ± 0.1 |
| Lung | 1.4 ± 0.1 | 3.1 ± 0.7 * | 3.7 ± 0.8 * | 2.7 ± 0.7 * |
| Heart | 0.89 ± 0.04 | 0.82 ± 0.04 | 0.82 ± 0.02 | 0.76 ± 0.05 |
| Liver | 8.3 ± 0.2 | 9.0 ± 0.4 | 9.9 ± 0.7 * | 9.9 ± 0.4 * |
| Kidney | 1.7 ± 0.1 | 1.6 ± 0.1 | 1.5 ± 0.1 | 1.6 ± 0.1 |
| Spleen | 0.57 ± 0.04 | 0.55 ± 0.02 | 0.52 ± 0.04 | 0.44 ± 0.02 * |
| Thymus | 0.47 ± 0.09 | 0.49 ± 0.05 | 0.49 ± 0.03 | 0.36 ± 0.04 |
| Ovaries | 0.16 ± 0.03 | 0.15 ± 0.01 | 0.16 ± 0.01 | 0.15 ± 0.01 |
| Uterus | 0.79 ± 0.10 | 0.71 ± 0.11 | 0.82 ± 0.16 | 0.71 ± 0.09 |
