# Supplementary material for: Pathophysiological Consequences of a Break in S1P1-Dependent Homeostasis of Vascular Permeability Revealed by S1P1 Competitive Antagonism
Source: PLoS One. 2016 Dec 22;11(12):e0168252. doi: 10.1371/journal.pone.0168252 (PMC5179015; doi:10.1371/journal.pone.0168252)
Supplement: S4 Table — Individual severity GRADES (- = none; 1 = minimal/very few and small; 2 = slight/few/small; 3 = moderate/moderate number and size; 4 = marked/many/ large) of the microscopic findings in lungs and heart of rats treated with NIBR-0213 or its vehicle. For each group, the Grades are indicated under the same the rat-order. (DOC) [file pone.0168252.s004.doc]

**S4 Table. Microscopic findings in lungs and heart of rats treated with NIBR-0213 over 2 weeks**

Individual severity GRADES (- =none; 1=minimal/very few and small; 2=slight/few/small; 3=moderate/moderate number and size; 4=marked/many/large) of the microscopic findings in lungs and heart of rats treated with NIBR-0213 or its vehicle. For each group, the Grades are indicated under the same the rat-order.

|  | **Vehicle** | | **30 mg/kg QD** | | **100 mg/kg QD** | | **300 mg/kg QD** | |
| --- | --- | --- | --- | --- | --- | --- | --- | --- |
|  | **M** | **F** | **M** | **F** | **M** | **F** | **M** | **F** |
| **n** | **5** | **5** | **4** | **5** | **5** | **5** | **5** | **5** |
| **Treatment related deaths** | **0** | **0** | **0** | **0** | **0** | **0** | **0** | **0/0/1/0/0** |
| **Severity grades in LUNGS:** |  |  |  |  |  |  |  |  |
| **Edema, alveolar** | **-** | **-** | **-/2/-/-** | **-/3/-/3/-** | **-/-/-/2/-** | **3/-/-/-/-** | **-** | **-/-/3/3/-** |
| **Hemorrhage, alveolar/interstitial** | **-/1/-/-/-** | **-** | **1/2/-/-** | **-/-/-/1/-** | **-/1/-/1/1** | **-/-/1/-/-** | **-/1/1/-/-** | **-** |
| **Necrosis, alveolar** | **-** | **-** | **1/1/1/-** | **-/2/1/2/1** | **2/1/-/2/2** | **1/-/1/1/1** | **-** | **-/-/1/2/1** |
| **Thickening, alveolar** | **-** | **-** | **1/2/1/2** | **1/2/1/2/1** | **2/2/1/2/2** | **2/1/2/2/1** | **1/1/1/2/1** | **1/2/2/2/1** |
| **Macrophages, alveolar** | **-** | **-** | **2/4/2/2** | **2/4/2/4/2** | **3/3/2/4/3** | **4/2/4/4/1** | **1/1/1/3/2** | **2/3/4/3/2** |
| **Fibrotic foci** | **-** | **-** | **1/1/-/1** | **-/1/-/1/-** | **3/3/-/1/1** | **2/1/1/1/-** | **-/-/-/2/-** | **-/1/2/2/-** |
| **Severity grades in HEARTS** |  |  |  |  |  |  |  |  |
| **Edema, Perivascular** | **-** | **-** | **2/2/1/-** | **1/2/1/2/1** | **1/1/1/1/1** | **2/-/1/2/2** | **-/-/1/1/-** | **-/-/1/1/-** |
| **Degeneration, myocytes** | **-** | **-** | **-** | **-/2/-/2/-** | **-/2/-/-/1** | **2/-/1/2/-** | **-/-/-/2/-** | **-/-/1/2/-** |
| **Fibrosis, interstitial** | **-** | **-** | **-** | **-** | **-/1/-/-/-** | **2/-/-/-/-** | **-** | **-** |
